# Whipple Disease Presenting as Isolated Transverse Myelitis with Permanent Neurological Damage in a Patient with Systemic Lupus Erythematosus: A Case Report of a Difficult Diagnosis with a Literature Review

**DOI:** 10.3390/idr16020022

**Published:** 2024-03-19

**Authors:** Carolina Saffioti, Marta Nebiolo, Roberta Caorsi, Alessio Mesini, Mariasavina Severino, Giacomo Brisca, Elio Castagnola, Marco Gattorno

**Affiliations:** 1 Pediatric Infectious Diseases Unit, IRCCS Istituto Giannina Gaslini, 16147 Genoa, Italy; carolinasaffioti@gaslini.org (C.S.); alessiomesini@gaslini.org (A.M.); eliocastagnola@gaslini.org (E.C.); 2 Department of Neuroscience, Rehabilitation, Ophthalmology, Genetics, Maternal and Child Health (DINOGMI), University of Genoa, 16132 Genoa, Italy; 5728549@studenti.unige.it; 3 Rheumatolgy and Autoinflammatory Diseases Unit, IRCCS Istituto Giannina Gaslini, 16147 Genoa, Italy; robertacaorsi@gaslini.org (R.C.); marcogattorno@gaslini.org (M.G.); 4 Neuroradiology Unit, IRCCS Istituto Giannina Gaslini, 16147 Genoa, Italy; mariasavinaseverino@gaslini.org; 5 Paediatric and Neonatal Intensive Care Unit, IRCCS Istituto Giannina Gaslini, 16147 Genoa, Italy

**Keywords:** *Tropheryma whipplei*, Whipple disease, systemic lupus erythematosus, immunosuppressive therapy, neuroimaging

## Abstract

We describe an atypical case of Whipple disease exclusively involving the spinal cord in an adolescent receiving immunosuppressive therapy for systemic lupus erythematosus. The diagnosis was particularly difficult since lupus and Whipple disease can present similar clinical features and the patient’s prolonged contact with sewage was initially not mentioned. A literature review of the clinical, imaging, diagnostic, and therapeutic challenges of Whipple disease is also performed.

## 1. Introduction

Whipple disease (WD) is a rare, chronic, systemic infection caused by Tropheryma whipplei (TW) [1,2]. Its main clinical features are abdominal pain, diarrhea, weight loss, and arthralgia, but cardiac, pulmonary, and neurological symptoms can also be present [3]. The central nervous system (CNS) is involved in 90% of cases of WD, but neurological manifestations are evident in only 10–43% [2,3], and are mainly represented by cognitive impairment, psychiatric dysfunction, sleep disturbances, oculo-masticatory myorhythmia, oculo-facio-skeletal myorhythmia, seizures, and ataxia, while medullary manifestations are rare, and few data are present in the literature [4,5,6,7,8,9,10,11,12,13,14,15,16].

Systemic lupus erythematosus (SLE) is an autoimmune disease that involves multiple organs; since the nervous system is an important target of immune-mediated damage, SLE often results in a complex spectrum of neurological syndromes [17]. The diagnostic criteria of Neurolupus are very challenging, but they broadly distinguish between complications affecting the central nervous system (CNS) and peripheral nervous system (PBS). The recognized clinical neurological syndromes are stroke, small vessel disease, seizures, myelopathy, meningitis movement disorders, demyelinating syndrome, headache, psychiatric disease, cognitive dysfunction (CNS) and inflammatory neuromuscular disease, peripheral neuropathy, and cranial neuropathy (PNS) [18]. Nervous system involvement is a major determinant of quality of life [19]; however, establishing a causal association between neurological symptoms and SLE is challenging [20]. Neurological manifestations can occur at any stage of the disease, and they are present in about 5% of patients with SLE [21].

Acute transverse myelitis (ATM) is a rare neurological syndrome that includes motor, sensory, and autonomic dysfunctions and can result in serious neurological disabilities in one-third of cases [22,23]. In the majority of cases, ATM is classified as idiopathic (considered to be an autoimmune process triggered by previous infection and/or vaccinations), while in a minority of cases, there is a clear etiology that can be infectious (due to viral, bacterial, fungal, or parasitic infection) or non-infectious in the context of a systemic inflammatory disease or multifocal CNS disease [22].

Here, we describe a case of WD, with unique involvement of the spinal cord, in a patient affected by systemic lupus erythematosus (SLE) with literature data on the epidemiological, clinical, diagnostic, and therapeutic features.

## 2. Case Report

A 14-year-old girl presented at IRCCS Istituto Giannina Gaslini Children’s Hospital, Genoa (Italy), with acute onset of diarrhea, a low-grade fever, headache, and asthenia followed by vomitus, lumbar pain, and severe lower limbs hyposthenia. The patient had been followed-up at our Institution for one year for systemic lupus erythematosus (SLE), which was well controlled with 100 mg azathioprine per day, 300 mg hydroxychloroquine per day, and low-dose steroids (22.5 mg prednisone per day).

**Figure 1:**
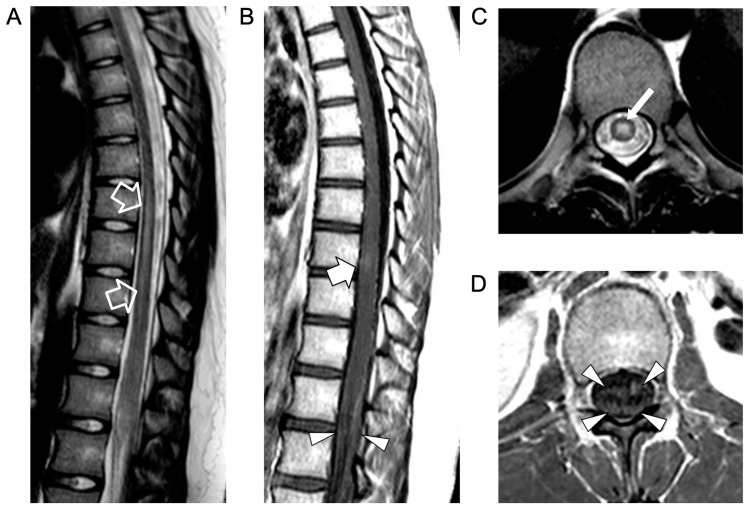
Contrast-enhanced spinal MRI performed at clinical onset. Sagittal T2-weighted (**A**) and post-contrast T1-weighted (**B**) images; axial T2-weighted (**C**) and post-contrast T1-weighted (**D**) images. There is a T2 hyperintensity in the central portion of the spinal cord (empty arrows in (**A**) and thin arrow in (**C**)) in keeping with an acute transverse myelitis extending from D7 to D9, associated with a faint area of contrast enhancement at the D8 level (thick arrow). Note the contrast enhancement of the anterior and posterior cauda equina nerve roots (arrowheads) indicative of myeloradiculitis.

An urgent spinal magnetic resonance imaging (MRI) scan showed acute transverse myelitis extending from D7 to D10, associated with contrast enhancement of the anterior and posterior roots of the cauda equina, indicative of myeloradiculitis (Figure 1).

The serum inflammatory indices were mildly increased with normal leukocyte counts, positive antinuclear antibody test (title 1:320, speckled), and anti-DNA antibodies. Cerebrospinal fluid (CSF) was crystal clear, with normal pressure. CSF analysis revealed an increase in white blood cells (1250 cells/μL) with a prevalence of polymorphonucleated protein (91 mg/dL) and low glucose levels (30 mg/dL). Culture for common bacteria and fungi was negative and specific polymerase chain reaction (PCR) for *N. meningitidis*, *S. pneumoniae*, parvovirus B19, CMV, EBV, HSV1-2, HHV6, and *Mycoplasma pneumoniae* were not detected. Anti-CMV, EBV, coxackievirus, echovirus, and parvovirus B19 antibodies were negative or compatible with previous infection.

**Figure 2:**
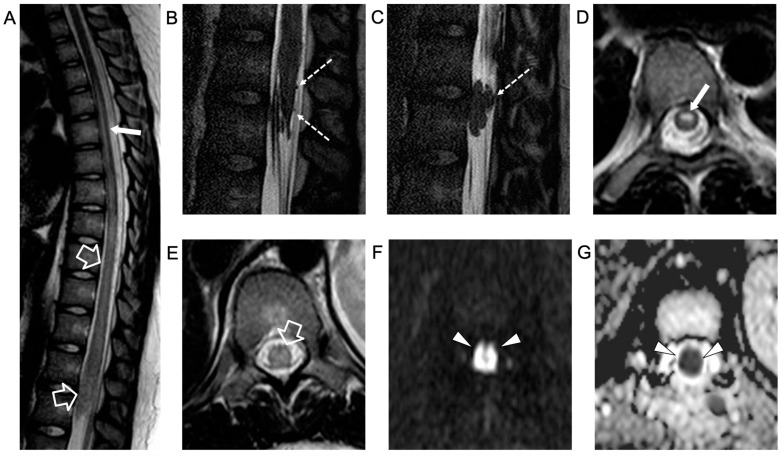
Contrast-enhanced spinal MRI performed 5 days after the clinical onset. Sagittal T2-weighted (**A**) and 3D driven equilibrium (DRIVE) (**B**,**C**) images; axial T2-weighted (**D**,**E**) images and diffusion-weighted image (**F**) with corresponding ADC map (**G**). There is a caudal extension of the T2 signal alterations involving the conus medullaris (empty arrows). Bilateral, symmetrical circular foci of high T2 signal are visible in the anterior horns of the spinal cord (i.e., owl-eyes sign) in the dorsal segment with a cranial extension to the D1 level (thin arrows). The spinal cord lesions are characterized by high signal on diffusion-weighted images with reduced ADC values (arrowheads) in keeping with a spinal cord infarction. In addition, there are several non-enhancing intradural extramedullary lesions along the cauda equina nerve roots and conus medullaris surface (dashed arrows).

The electrophysiological study showed a low amplitude of somatosensory evoked potentials (SSEPs) in the lower limbs with the absence of F wave. Empirical therapy with ceftriaxone twice daily, 750 g acyclovir three times a day, 2 g/kg immunoglobulins, and 1 g/dose methylprednisolone (×4 bolus) was administered with a decrease in lumbar pain but no improvement in the strength of the lower limbs. A second spinal MRI, performed 5 days later, showed caudal extension of the acute transverse myelitis involving the conus medullaris, with more pronounced nerve root contrast enhancement, associated with an anterior spinal cord infarction (Figure 2)

**Figure 3:**
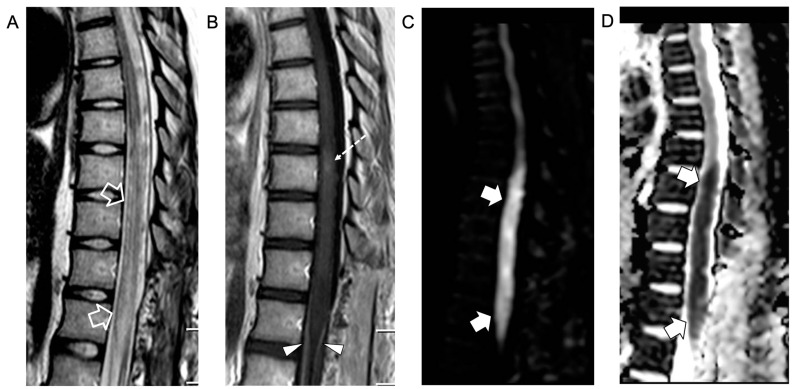
Contrast-enhanced spinal MRI performed 6 weeks after clinical onset. Sagittal T2-weighted (**A**), post-contrast T1-weighted (**B**), and diffusion-weighted (**C**) images with corresponding ADC map (**D**). The swelling and T2 signal alterations in the lower dorsal spinal cord and conus medullaris are worsened (empty arrows) with persistence of the focal intramedullary contrast enhancement (dashed arrow) and cauda equina nerve root contrast enhancement (arrowheads). Note that there is a new acute ischemic infarct at the level of the conus medullaris (thick arrows).

An intradural extramedullary lobulated lesion was also noted at the level of the conus medullaris, with several similar small nodular lesions spreading along the cauda equina nerve roots and conus medullaris surface (Figure 2). A brain MRI demonstrated small subcortical gliotic changes in the right temporal lobe that remained stable in follow-up studies. Chest computed tomography (CT) and whole-body MRI were negative, as well as spinal digital subtraction angiography. A new CSF examination was performed: no atypical cells were detected but immunochemical tests showed barrier damage in the absence of oligoclonal bands. New microbiological tests and PCR on CSF and blood excluded some infective etiologies, in particular the presence of *Cryptococcus*, *Aspergillus*, *Mycobacterium tuberculosis*, *Borrelia*, *Yersinia*, *Toxoplasma*, and *Bartonella*. Antimicrobial therapy was shifted to 600 mg teicoplanin per day, subsequently substituted with 3 g ampicillin four times a day. For the suspicion of neoplastic disease, a biopsy of the extramedullary lesion was performed, revealing an ischemic lesion with perivascular inflammatory infiltrates and phagocytosis of uncertain significance, suggestive of an unspecified infective lesion. While waiting for the histological analysis, a second infusion of immunoglobulin, oral cyclophosphamide, and steroids was started for the suspicion of SLE-related transverse myelitis. An immediate postoperative spinal MRI performed 3 weeks after the onset revealed complete removal of the intradural extramedullary mass, while the spinal cord lesion and nerve root involvement were stable. The clinical picture worsened with progressive paraplegia and anesthesia of the lower limbs, urinary incontinence, and deterioration of neurophysiological findings. During the following weeks, plasmapheresis, cyclophosphamide, and immunoglobulin were administered and ampicillin was continued. Spinal MRI performed 6 weeks after clinical onset and showed a new acute ischemic lesion involving the inferior dorsal spinal cord and conus medullaris and additional small nodular lesions along the conus medullaris surface (Figure 3).

Considering the radiological progression and despite the negative microbiological results, therapy was modified with ceftriaxone, doxycycline, and plaquenil administration. PCR for the screening of bacterial ribosomal RNA (PCR16S) performed by the Standford University Laboratory of Microbiology had negative results. Cyclophosphamide was stopped and low-dose mycophenolate mofetil associated with low-dose oral steroids was started to control the underlying SLE.

A re-evaluation of the case with several national and international experts was performed. The staining of the biopsy revealed a PAS-positive macrophage infiltration of the extramedullary lesion. Reviewing the patient’s medical history, the parents revealed that, before the onset of symptoms, a sewage pipe had broken in their garden with infiltration of sewer water in the walls of their home, lasting for some months before disease onset. Therefore, PCR for *Tropheryma whipplei* on a sample of the biopsied lesion was sent to the Department of Medical Laboratory Sciences and Infectious Disease of the Gemelli University Hospital, Rome (Italy), and showed positive results. PCR on saliva and stools for the same pathogen was negative. Specific antibiotic therapy with ceftriaxone, doxycycline, and trimethoprim-sulphamethoxazole was started. Mycophenolate was maintained with complete control of the underlying SLE.

**Figure 4:**
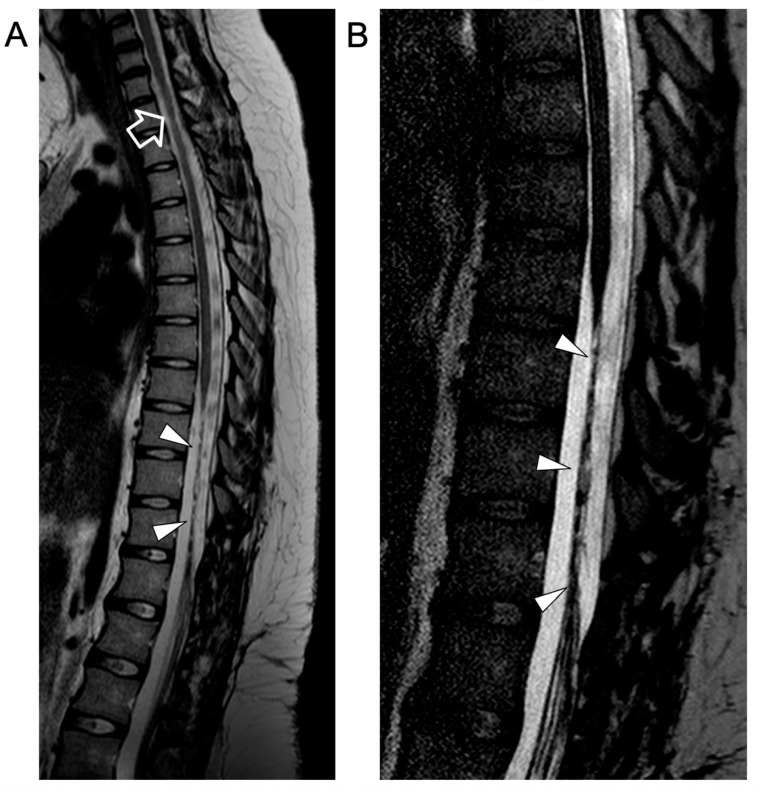
Contrast-enhanced spinal MRI performed at the last follow-up, several months after clinical onset. Sagittal T2-weighted (**A**) and 3D driven equilibrium (DRIVE) (**B**) images reveal a focal area of mild spinal cord thinning at the superior dorsal level (empty arrow) and an extended segment of severe spinal cord atrophy in the inferior dorsal spinal level (arrowheads).

In the following months, repeated MRI studies revealed progressive disappearance of the intradural extramedullary lesions, regression of cauda equina contrast enhancement, and chronic evolution of the spinal cord lesions. At the last follow-up, performed at 18 years of age, the neurological examination was unchanged, showing complete paralysis of the lower limbs. Spinal MRI revealed stable atrophy and gliotic changes of the affected dorsal spinal cord and conus medullaris (Figure 4).

Only weeks after the onset of clinical signs, the parents claimed the presence of sewage water infiltration in their house walls, and we suppose that this could have been the source of the infection.

## 3. Literature Review and Discussion

We described a case of difficult-to-diagnose spinal cord involvement by TW in an adolescent with SLE. The literature data on the epidemiological, clinical, diagnostic, and therapeutic features of WD were reviewed in the wake of this very peculiar observation.

Epidemiology: TW is a Gram-positive, PAS-positive, rod-shaped bacterium belonging to the Actinomycetes group [4] and is present in soil, seawater sediment, and sewerage systems [1,24,25]. In humans, TW can be isolated from duodenal biopsy, the stool, and the saliva of affected individuals or asymptomatic carriers [1,24,25]. Humans can be colonized by TW from the environment (for example, drinking contaminated water) or with a possible inter-human oral–fecal transmission. Relatives of patients with chronic WD (CWD) have a higher risk of becoming carriers of TW but it is not clear if there is an inter-human transmission or if they are exposed to the same source of infection [26]. TW has been found in 15% of the stool of children aged 2 to 4 years with gastroenteritis [27]. In Europe, TW is isolated in fecal samples of asymptomatic individuals in 1–8% of cases, reaching 12–25% among categories at risk, such as sewer workers, houseless people, and people with HIV [25,28,29,30,31,32,33,34,35,36,37,38]. WD is described worldwide with a 1/1,000,000 prevalence, with variable geographic distribution [24,29], and typically affects middle-aged men [2,30]. In Italy, the prevalence of WD is reported to be 3/1,000,000 [39], while the overall intestinal colonization rate is 6.7%, rising to 12.7% in children [40]. In most cases, TW is eliminated by the immune system without the development of any symptoms or after a self-limiting infection with the acquisition of humoral and cellular immunity [2,41]. However, in the presence of predisposing factors, such as HLA-DRB1*13 and DQB1*06 alleles that impair the normal presentation of antigens, chronic infection may develop [42]. Immunologic defects can play an important role in the pathogenesis of WD, especially when involving the macrophages, which can phagocytes in TW but are not able to degrade them [43], T cells, and humoral immune response [43,44,45,46,47,48]. These immune defects seem to be specific for TW since patients are not predisposed to other infections. HIV disease or medical immunosuppression (e.g., given for the treatment of unclear arthropathy) can be a trigger for the onset of clinical manifestation of WD in predisposed or colonized individuals [49].

Clinical picture: Asymptomatic TW carriers have been described [24,25,50,51], while WD is a heterogenic, multisystem disease that can present as [52,53] acute transient disease with fever and diarrhea [24,28,38,54]; localized infection, e.g., endocarditis or central nervous system disease [3,55,56,57,58]; or classic systemic disease characterized by a broad spectrum of clinical signs and symptoms, including weight loss, arthralgia, and diarrhea [2]. Sometimes these symptoms are misinterpreted, and patients are treated for rheumatologic diseases with immune suppressants that can accelerate the appearance of the systemic phase [51].

CNS disease is the most severe manifestation of WD, which is frequently overlooked, and occurs in 10–43% of patients [2,3,5,11,13,57]. Post mortem brain biopsies show the presence of TW in 90% of cases [5]. Neurologic symptoms can mimic many other neurological conditions. The most frequent are cognitive disorders, such as dementia, psychiatric dysfunction, or behavior changes, which are present in 61–71% of cases [3,5,13,59]. Oculo-facial-skeletal myorhythmia (OSM) and oculo-masticatory myorhythmia (OMM) are present in 20% of cases and are strongly suggestive of WD [60]. Hypothalamic involvement manifests as sleep disturbances like hypersomnia or severe insomnia [61], hyperphagia, polyuria, polydipsia, and libido disorders [62]. Other CNS manifestations are cerebellar ataxia [63], seizure and headache [12,64], pyramidal and extrapyramidal symptoms, supranuclear ophthalmoplegia [3,5,14], stroke [56], encephalitis and meningitis [14,59,65], and obstructive hydrocephalus [66]. Signs of sensory–motor myelopathy have been reported in rare cases of spinal cord involvement [3,6,8,9,13], while peripheral involvement is usually related to secondary malabsorption and nutritional deficits [5]. Notably, CNS disease can appear as a neurological relapse of treated classical WD, as a manifestation of classical WD, or as an isolated identity without histological evidence of intestinal disease [2,3,12].

While neurologic complications of WD are common, acute transverse myelitis (AMT) is a rare complication. As previously said, there are many different causes of transverse myelitis, and they are typically classified as either idiopathic or disease-associated.

The infectious etiology affects about 12% of patients and this is why, despite being a rare condition, it needs to be considered among the top differential diagnoses, given the importance of timely recognition and initiation of therapy [24].

Different clinical manifestations have been associated with immunosuppressive drugs (e.g., tumor necrosis factor blockers), often started after a misdiagnosis of rheumatic arthritis [49,61]. Immunosuppressive treatments cause an immunologically impaired state, influencing the course of the disease. Therapy alters the intestinal barrier function, accelerating the onset of classic WD and fostering a bacterial load compared to immunocompetent individuals, ending in worse gastrointestinal manifestations, endocarditis, spondylitis, or CNS involvement [57,67].

Sometimes WD and autoimmune disease manifestations are mixed, and this can make the diagnosis more difficult [68]. WD in association with malignancies (e.g., lymphomas) and prior chemotherapy has also been described [57]. Notably, WD in association with immunosuppression increases the risk of immune reconstitution inflammatory syndrome (IRIS) after the start of antibiotic therapy [69,70,71].

Diagnosis: WD is usually diagnosed by duodenal biopsies [2,52]. Pale yellow intestinal mucosa alternating with erythematous, erosive mucosa with blunted villi and engorged lymphatic vessels can be observed [53,57]. The presence of macrophages containing PAS-positive materials in the lamina propria of the duodenum (but also the stomach, jejune, or ileus) is suggestive of WD, and in most cases, it is positive even if there are no significant intestinal manifestations [53,57]. Noteworthy PAS-positive materials in macrophages can be found years after the start of adequate therapy [53] and an increase in PAS-positive materials may be an early relapse sign. In the suspicion of WD, multiple biopsies must be obtained because of the possible patchy distribution of the lesions [1,53].

According to the clinical picture, biopsies may be obtained from other tissues, such as the CNS, cardiac valve, synovia, or lymph nodes; however, the presence of PAS-positive lesions in these tissues has a limited diagnostic value [72]. Remarkably, the presence of PAS-positive materials in intestinal specimens is indicative but not pathognomonic of WD, and other infections causing similar histological features, such as mycobacterial infection, have to be excluded [1]. Another histological finding in WD is the presence of non-caseating, epithelioid cell granulomas in gastrointestinal and lymphatic samples. Differential diagnosis with Mycobacterium avium complex, Bacillus cereus, Histoplasma, Corynebacterium, Rhodococcus, and invasive fungal diseases, especially in immunocompromised patients, have to be performed [2,72]. Polymerase chain reaction (PCR) and/or immunohistochemistry (IHC) are recommended to confirm diagnosis [1,2,72]. PCR-based diagnosis can be made on sterile tissue samples that are not in contact with the environment, such as the CSF, synovial fluid, ascites, humor vitreous or pleural effusion, and CNS biopsies [1,57,72]. In addition, performing PCR on the CSF, even in the absence of neurological signs, is recommended because asymptomatic CNS involvement is present in 50% of cases of WD [26,53,72,73].

To avoid the risk of contamination, performing at least two PCR tests on primers obtained from two different genes or the use of IHC is indicated, particularly in atypical cases [26,53,72,73]. Western blot serology has been proposed to discriminate asymptomatic, PCR-positive carriers, who generally have an important immune response, from classical WD, in which immune response is low. However, this test is not widely available yet [1,26]. Recently, TW was detected in urine samples of untreated patients with classical WD or localized WD [74,75]. However, the authors reiterate the importance of invasive sampling for the diagnosis of WD; therefore, a urine search of TW can be an easy-to-perform first screening in the suspicion of WD or in patients with unclear rheumatic diseases [74,75].

No imaging test is specific for WD. 18-FDG-PET has been proposed for initial evaluation and follow-up [76]. Brain CT or MRI are recommended in cases of suspect CNS involvement, but lesions are not specific [3]. Neuroimaging is not specific in WD. However, two recurrent patterns have been described on brain MRI: (i) multiple, nodular, contrast-enhancing lesions with perilesional vasogenic edema, mainly located in the frontal and temporal lobes, basal ganglia, periventricular white matter, cingulum, hypothalamus, brainstem, and cerebellum (with peculiar involvement of the middle cerebellar peduncles) [11,77]; (ii) a single cerebral lesion with mass effect and a “tumor-like” appearance [64,78,79]. Leptomeningeal involvement and/or ependymal contrast enhancement may be present, as well as obstructive hydrocephalus. Associated spinal cord involvement is rare [6,14] and isolated spinal cord involvement is even rarer, with only a few adult cases described in the literature [3,6,8,9,13]. Notably, in all cases of isolated spinal cord involvement, lesions were observed in the cervical or cervicothoracic tract [6,8,9,10]. Interestingly, a remitting–relapsing course of myelopathy is described in some of these cases [6,8]. Finally, in rare cases, neuroimaging may be normal even in the presence of neurological symptoms [14,62].

Therapy: The best therapeutic approach and duration of treatment for WD are still debated. Standard therapy is a two-week induction phase of ceftriaxone or meropenem followed by maintenance with cotrimoxazole for 1 year [1,73]. A short-term maintenance phase of 3 months has been suggested as more effective than a longer one [80], but subsequent studies have reported cases of relapse, including CNS involvement, during therapy with cotrimoxazole [75,79,81]. An alternative therapeutic scheme with doxycycline plus hydroxychloroquine for 1 year followed by life-long prophylaxis with doxycycline has been proposed [59,82]. A high therapeutic efficacy of ceftriaxone plus cotrimoxazole has also been observed in the first year of treatment followed by life-long prophylaxis with doxycycline [83]. Notably, IRIS is the most important, life-threatening complication during the treatment of WD. Typically, it occurs in patients previously (even years before) treated with immunosuppressants and it is due to an uncontrolled reconstitution of the immune system [69,70,71]. IRIS must be suspected if inflammatory symptoms recur after effective treatment and must be promptly treated with corticosteroids.

Our patient had an atypical presentation of WD involving only the spinal cord and the diagnostic hypothesis was raised after discussing the case with numerous national and international colleagues, and after knowing about the patient’s prolonged contact with sewage material. After a literature review, the diagnosis of SLE was also questioned since the initial symptoms, interpreted as the onset of a rheumatic disease, could have been the first signs of WD. However, even in the absence of kidney involvement, the patient’s onset, symptoms, and signs fulfilled the diagnostic criteria of SLE (SLICC-2012) [84], i.e., arthromyalgia, low-grade and persistent fever, malar rash, an increase in the erythro-sedimentation rate, lymphopenia, the reduction of complement factors (C3 and C4), high titer antinuclear antibodies (ANAs), and anti-double-stranded DNA (ds) antibodies (Abs ds-DNA). Clinical and laboratory features responded dramatically to the standard treatment with hydroxychloroquine, azathioprine, and low-dose steroids for more than one year. We therefore hypothesized that immune-suppressive therapies together with the environmental exposure to sewage and a possible genetic predisposition caused the onset of WD in the patient. Spinal cord involvement in WD is very rare, but except for minimal gastrointestinal symptoms, it was the only sign of the disease in our patient. The atypical presentation and the underlying autoimmune condition made a challenging diagnosis even more difficult.

## 4. Conclusions

WD is a rare but important differential diagnosis in patients with chronically progressive or relapsing–remitting isolated myelitis, also in pediatrics. Based on our experience, we suggest considering and excluding WD in cases with unknown medullary lesions, especially in immunocompromised patients.

## Data Availability

No new data were created.
